# Clinician perspectives and recommendations regarding design of clinical prediction models for deteriorating patients in acute care

**DOI:** 10.1186/s12911-024-02647-4

**Published:** 2024-09-02

**Authors:** Robin Blythe, Sundresan Naicker, Nicole White, Raelene Donovan, Ian A. Scott, Andrew McKelliget, Steven M McPhail

**Affiliations:** 1 Australian Centre for Health Services Innovation and Centre for Healthcare Transformation, School of Public Health and Social Work, Faculty of Health, Queensland University of Technology, 60 Musk Ave, Kelvin Grove, Brisbane, QLD 4059 Australia; 2 Princess Alexandra Hospital, Metro South Health, Woolloongabba, QLD Australia; 3 Queensland Digital Health Centre, Faculty of Medicine, University of Queensland, Brisbane, QLD Australia; 4 Digital Health and Informatics Directorate, Metro South Health, Woolloongabba, QLD Australia

**Keywords:** Clinical prediction models, Clinical decision support systems, Early warning score, Clinical deterioration, Clinical decision-making

## Abstract

**Background:**

Successful deployment of clinical prediction models for clinical deterioration relates not only to predictive performance but to integration into the decision making process. Models may demonstrate good discrimination and calibration, but fail to match the needs of practising acute care clinicians who receive, interpret, and act upon model outputs or alerts. We sought to understand how prediction models for clinical deterioration, also known as early warning scores (EWS), influence the decision-making of clinicians who regularly use them and elicit their perspectives on model design to guide future deterioration model development and implementation.

**Methods:**

Nurses and doctors who regularly receive or respond to EWS alerts in two digital metropolitan hospitals were interviewed for up to one hour between February 2022 and March 2023 using semi-structured formats. We grouped interview data into sub-themes and then into general themes using reflexive thematic analysis. Themes were then mapped to a model of clinical decision making using deductive framework mapping to develop a set of practical recommendations for future deterioration model development and deployment.

**Results:**

Fifteen nurses (*n* = 8) and doctors (*n* = 7) were interviewed for a mean duration of 42 min. Participants emphasised the importance of using predictive tools for supporting rather than supplanting critical thinking, avoiding over-protocolising care, incorporating important contextual information and focusing on how clinicians generate, test, and select diagnostic hypotheses when managing deteriorating patients. These themes were incorporated into a conceptual model which informed recommendations that clinical deterioration prediction models demonstrate transparency and interactivity, generate outputs tailored to the tasks and responsibilities of end-users, avoid priming clinicians with potential diagnoses before patients were physically assessed, and support the process of deciding upon subsequent management.

**Conclusions:**

Prediction models for deteriorating inpatients may be more impactful if they are designed in accordance with the decision-making processes of acute care clinicians. Models should produce actionable outputs that assist with, rather than supplant, critical thinking.

**Supplementary Information:**

The online version contains supplementary material available at 10.1186/s12911-024-02647-4.

## Highlights

• This article explored decision-making processes of clinicians using a clinical prediction model for deteriorating patients, also known as an early warning score.

• Our study identified that the clinical utility of deterioration models may lie in their assistance in generating, evaluating, and selecting diagnostic hypotheses, an important part of clinical decision making that is underrepresented in the prediction modelling literature.

• Nurses in particular stressed the need for models that encourage critical thinking and further investigation rather than prescribe strict care protocols.

**Supplementary Information:**

The online version contains supplementary material available at 10.1186/s12911-024-02647-4.

## Background

The number of ‘clinical prediction model’ articles published on PubMed has grown rapidly over the past two decades, from 1,918 articles identified with these search terms published in 2002 to 26,326 published in 2022. A clinical prediction model is defined as any multivariable model that provides patient-level estimates of the probability or risk of a disease, condition or future event [1–3]. 

Recent systematic and scoping reviews report a lack of evidence that clinical decision support systems based on prediction models are associated with improved patient outcomes once implemented in acute care [4–7]. One potential reason may be that some models are not superior to clinical judgment in reducing missed diagnoses or correctly classifying non-diseased patients [8]. While improving predictive accuracy is important, this appears insufficient for improving patient outcomes, suggesting that more attention should be paid to the process and justification of how prediction models are designed and deployed [9, 10]. 

If model predictions are to influence clinical decision-making, they must not only demonstrate acceptable accuracy, but also be implemented and adopted at scale in clinical settings. This requires consideration of how they are integrated into clinical workflows, how they generate value for users, and how clinicians perceive and respond to their outputs of predicted risks [11, 12]. These concepts are tenets of user-centred design, which focuses on building systems based on the needs and responsibilities of those who will use them. User-centred decision support tools can be designed in a variety of ways, but may benefit from understanding the characteristics of the users and the local environment in which tools are implemented, [13] the nature of the tasks end-users are expected to perform, [14] and the interface between the user and the tools [15]. 

### Prediction models for clinical deterioration

A common task for prediction models integrated into clinical decision support systems is in predicting or recognising clinical deterioration, also known as early warning scores. Clinical deterioration is defined as the transition of a patient from their current health state to a worse one that puts them at greater risk of adverse events and death [16]. Early warning scores were initially designed to get the attention of skilled clinicians when patients began to deteriorate, but have since morphed into complex multivariable prediction models [17]. As with many other clinical prediction models, early warning scores often fail to demonstrate better patient outcomes once deployed [4, 18]. The clinical utility of early warning scores likely rests on two key contextual elements: the presence of uncertainty, both in terms of diagnosis and prognosis, and the potential for undesirable patient outcomes if an appropriate care pathway is delayed or an inappropriate one is chosen [19]. 

The overarching goal of this qualitative study was to determine how prediction models for clinical deterioration, or early warning scores, could be better tailored to the needs of end-users to improve inpatient care. This study had three aims. First, to understand the experiences and perspectives of nurses and doctors who use early warning scores. Second, to identify the tasks these clinicians performed when managing deteriorating patients, the decision-making processes that guided these tasks, and how these could be conceptualised schematically. Finally, to address these tasks and needs with actionable, practical recommendations for enhancing future deterioration prediction model development and deployment.

## Methods

**Fig. 1:**
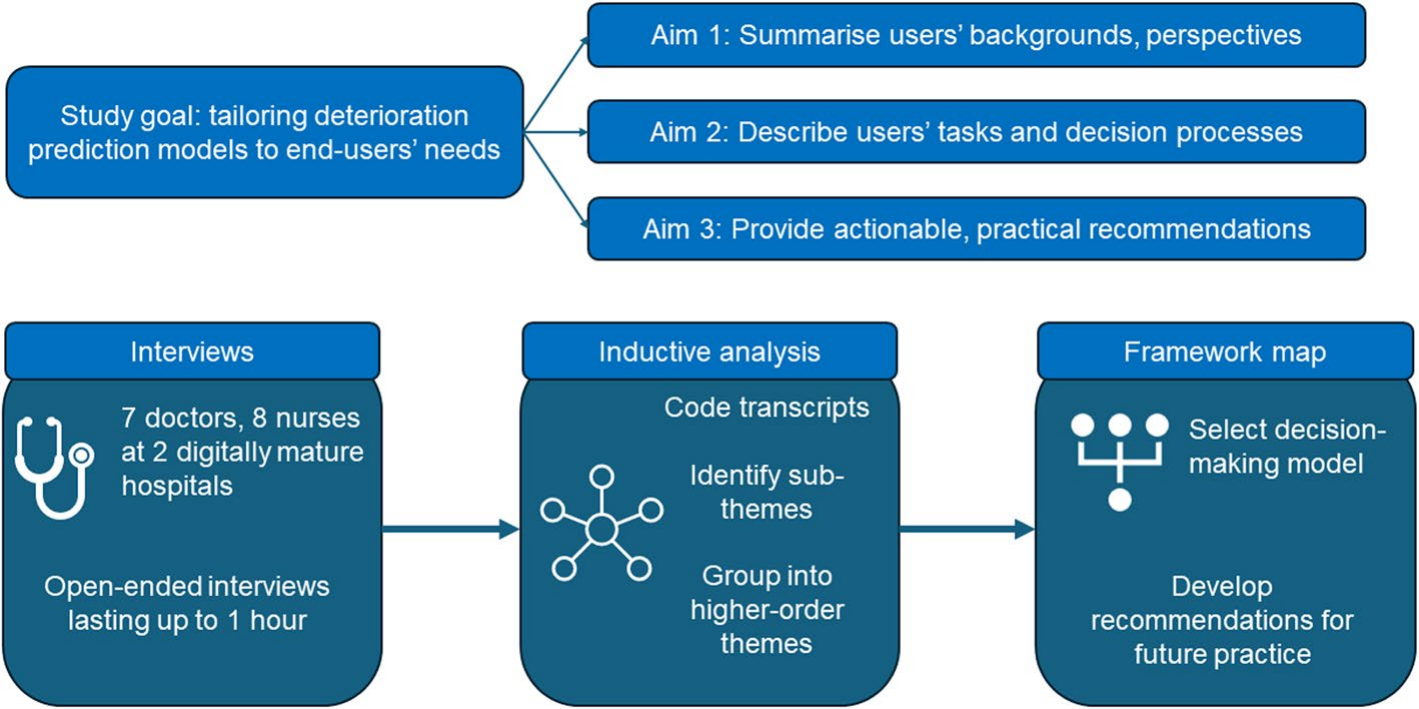
Schema of study goal, aims and methods

To achieve our study aims, we conducted semi-structured interviews of nurses and doctors at two large, digitally mature hospitals. We first asked clinicians to describe their backgrounds, perspectives, and experience with early warning scores to give context to our analysis. We then examined the tasks and responsibilities of participants and the decision-making processes that guided these tasks using reflexive thematic analysis, an inductive method that facilitated the identification of general themes. We then identified a conceptual decision-making framework from the literature to which we mapped these themes to understand how they may lead to better decision support tools. Finally, we used this framework to formulate recommendations for deterioration prediction model design and deployment. These steps are presented graphically in a flow diagram (Fig. 1).

### Setting

The study was conducted at one large tertiary and one medium-sized metropolitan hospital in Brisbane, Australia. The large hospital contained over 1,000 beds, handling over 116,000 admissions and approximately 150,000 deterioration alerts per year in 2019. Over the same period, the medium hospital contained 175 beds, handling over 31,000 admissions and approximately 42,000 deterioration alerts per year. These facilities had a high level of digital maturity, including fully integrated electronic medical records.

### Clinical prediction model for deteriorating patients

The deterioration monitoring system used at both hospitals was the Queensland Adult Deterioration Detection System (Q-ADDS) [20, 21]. Q-ADDS uses an underlying prediction model to convert patient-level vital signs from a single time of observation into an ordinal risk score describing an adult patient’s risk of acute deterioration. Vital signs collected are respiratory rate (breaths/minute), oxygen flow rate (L/minute), arterial oxygen saturation (percent), blood pressure (mmHg), heart rate (beats/minute), temperature (degrees Celsius), level of consciousness (Alert-Voice-Pain-Unresponsive) and increased or new onset agitation. Increased pain and urine output are collected but not used for score calculation [21]. The Q-ADDS tool is included in the supplementary material.

Vital signs are entered into the patient’s electronic medical record, either imported from the vital signs monitoring device at the patient’s bedside or from manual entry by nurses. Calculations are made automatically within Q-ADDS to generate an ordinal risk score per patient observation. Scores can be elevated to levels requiring a tiered escalation response if a single vital sign is greatly deranged, or if several observations are deranged by varying degrees. Scores range from 0 to 8+, with automated alerts and escalation protocols ranging from more frequent observations for lower scores to immediate activation of the medical emergency team (MET) at higher scores.

The escalation process for Q-ADDS is highly structured, mandated and well documented [21]. Briefly, when a patient’s vital signs meet a required alert threshold, the patient’s nurse is required to physically assess the patient and, depending on the level of severity predicted by Q-ADDS, notify the patient’s doctor (escalation). The doctor is then required to be notified of the patient’s Q-ADDS score, potentially review the patient, and discuss any potential changes to care with the nurse. Both nurses and doctors can escalate straight to MET calls or an emergency ‘code blue’ call (requiring cardiopulmonary resuscitation or assisted ventilation) at any time if necessary.

### Participant recruitment

Participant recruitment began in February 2022 and concluded in March 2023, disrupted by the COVID-19 pandemic. Eligibility criteria were nurses or doctors at each hospital with direct patient contact who either receive or respond, respectively, to Q-ADDS alerts. An anticipated target sample size of 15 participants was established prior to recruitment, based on expected constraints in recruitment due to clinician workloads and the expected length of interviews relative to their scope, as guided by prior research [22]. As the analysis plan involved coding interviews iteratively as they were conducted, the main justification for ceasing recruitment was when no new themes relating to the study objectives were generated during successive interviews as the target sample size was approached [23]. 

Study information was broadly distributed via email to nurses and doctors in patient-facing roles across hospitals. Nurse unit managers were followed up during regular nursing committee meetings to participate or assist with recruitment within their assigned wards. Doctors were followed up by face-to-face rounding. Snowball sampling, in which participants were encouraged to refer their colleagues for study participation, was employed whenever possible. In all cases, study authors explained study goals and distributed participant consent forms prior to interview scheduling with the explicit proviso that participation was completely voluntary and anonymous to all but two study authors (RB and SN).

### Interview process

We used a reflexive framework method to develop an open-ended interview template [24] that aligned with our study aims. Interview questions were informed by the non-adoption, abandonment, scale-up, spread and sustainability (NASSS) framework [25]. The NASSS framework relates the end-user perceptions of the technology being evaluated to its value proposition for the clinical situation to which it is being applied. We selected a reflexive method based on the NASSS for our study as we wanted to allow end-users to speak freely about the barriers they faced when using prediction models for clinical deterioration, but did not limit participants to discussing only topics that could fit within the NASSS framework.

Participants were first asked about their background and clinical expertise. They were then invited to share their experiences and perspectives with using early warning scores to manage deteriorating patients. This was used as a segue for participants to describe the primary tasks required of them when evaluating and treating a deteriorating patient. Participants were encouraged to talk through their decision-making process when fulfilling these tasks, and to identify any barriers or obstacles to achieving those tasks that were related to prediction models for deteriorating patients. Participants were specifically encouraged to identify any sources of information that were useful for managing deteriorating patients, including prediction models for other, related disease groups like sepsis, and to think of any barriers or facilitators for making that information more accessible. Finally, participants were invited to suggest ways to improve early warning scores, and how those changes may lead to benefits for patients and clinicians.

As we employed a reflexive methodology to allow clinicians to speak freely about their perspectives and opinions, answers to interview questions were optional and open-ended, allowing participants to discuss relevant tangents. Separate interview guides were developed for nurses and doctors as the responsibilities and information needs of these two disciplines in managing deteriorating patients often differ. Nurses are generally charged with receiving and passing on deterioration alerts, while doctors are generally charged with responding to alerts and making any required changes to patient care plans [4]. Interview guides are contained in the supplement.

Due to clinician workloads, member checking, a form of post-interview validation in which participants retrospectively confirm their interview answers, was not used. To ensure participants perceived the interviewers as being impartial, two study authors not employed by the hospital network and not involved in direct patient care (RB and SN) were solely responsible for conducting interviews and interrogating interview transcripts. Interviews were recorded and transcribed verbatim, then re-checked for accuracy.

### Inductive thematic analysis

Transcripts were analysed using a reflexive thematic methodology informed by Braun and Clarke [26]. This method was selected because it facilitated exploring the research objectives rather than being restricted to the domains of a specific technology adoption framework, which may limit generalisability [27]. Interviews were analysed over five steps to identify emergent themes.

1. Each interview was broken down into segments by RB and SN, where segments corresponded to a distinct opinion.
2. Whenever appropriate, representative quotes for each distinct concept were extracted.
3. Segments were grouped into sub-themes.
4. Sub-themes were grouped into higher-order themes, or general concepts.
5. Steps 1 through 4 were iteratively repeated by RB and supervised by SN.

As reflexive methods incorporate the experiences and expertise of the analysts, our goal was to extract any sub-themes relevant to the study aims and able to be analysed in the context of early warning scores, prediction models, or decision support tools for clinical deterioration. The concepts explored during this process were not exhaustive, but repeated analysis and re-analysis of participant transcripts helped to ensure all themes could be interpreted in the context of our three study aims: background and perspectives, tasks and decision-making, and recommendations for future practice.

### Deductive mapping to a clinical decision-making framework

Once the emergent themes from the inductive analysis were defined, we conducted a brief scan of PubMed for English-language studies that investigated how the design of clinical decision support systems relate to clinical decision-making frameworks. The purpose of this exercise was to identify a framework against which we could map the previously elicited contexts, tasks, and decision-making of end-users in developing a decision-making model that could then be used to support the third aim of formulating recommendations to enhance prediction model development and deployment.

RB and SN then mapped higher-order themes from the inductive analysis to the decision-making model based on whether there was a clear relationship between each theme and a node in the model (see Results).

Recommendations for improving prediction model design were derived by reformatting the inductive themes based on the stated preferences of the participants. These recommendations were then assessed by the remaining authors and the process repeated iteratively until authors were confident that all recommendations were concordant with the decision-making model.

## Results

### Participant characteristics

Our sample included 8 nurses and 7 doctors of varying levels of expertise and clinical specialties; further information is contained in the supplement. Compared to doctors, nurse participants were generally more experienced, often participating in training or mentoring less experienced staff. Clinical specialities of nurses were diverse, including orthopaedics, cancer services, medical assessment and planning unit, general medicine, and pain management services. Doctor participants ranged from interns with less than a year of clinical experience up to consultant level, including three doctors doing training rotations and two surgical registrars. Clinical specialties of doctors included geriatric medicine, colorectal surgery, and medical education.

### Interviews and thematic analysis

Eleven interviews were conducted jointly by RB and SN, one conducted by RB, and three by SN. Interviews were scheduled for up to one hour, with a mean duration of 42 min. Six higher-order themes were identified. These were: added value of more information; communication of model outputs; validation of clinical intuition; capability for objective measurement; over-protocolisation of care; and model transparency and interactivity (Table 1). Some aspects of care, including the need for critical thinking and the informational value of discerning trends in patient observations, were discussed in several contexts, making them relevant to more than one higher-order theme.

**Table 1:** Higher-order inductive themes and component sub-themes^a^

Inductive theme	Description	Component sub-themes
Added value of more information	Additional data that clinicians use for decision making	Omission of other quantifiable variables relevant to decision-making Nursing concern for patient Whole-person care
Model outputs	The delivery and format of prediction outputs	Output frequency and lack of relevance (alert fatigue) Arbitrary scoring mechanisms which do not generate probability (risk) estimates
Validation of clinical intuition	Model utility in confirming suspicions	Framing escalation conversations Added weight of evidence to generating/selecting hypotheses for causes of deterioration Potential to induce tunnel vision
Objective measurement	Model generation of objective outputs	Can correct for clinician bias Can assist with triage and prioritisation Measure of cumulative change over time (early detection) Potential for measurement error
Over-protocolisation of care	Staff adhering to a pre-specified stimulus-response interaction	Inhibits critical thinking of causes of deterioration and effects Reluctance to act if diverging from model recommendations Reduces mindful observation
Model transparency and interactivity	Clinician desire to query and understand prediction models	Importance of effect size and direction of predictor variables Colour-coding, visualisation Trends analysis Training to understand the model

^a^As identified by participants, briefly defined, including words commonly used by participants to describe the concept

### Added value of other information

Clinicians identified that additional data or variables important for decision making were often omitted from the Q-ADDS digital interface. Such variables included current medical conditions, prescribed medications and prior observations, which were important for interpreting current patient data in the context of their baseline observations under normal circumstances (e.g., habitually low arterial oxygen saturation due to chronic obstructive pulmonary disease) or in response to an acute stimulus (e.g., expected hypotension for next 4 to 8 h while treatment for septic shock is underway).

*“The trend is the biggest thing [when] looking at the data*,* because sometimes people’s observations are deranged forever and it’s not abnormal for them to be tachycardic*,* whereas for someone else*,* if it’s new and acute*,* then that’s a worry.”* – Registrar.

Participants frequently emphasised the critical importance of looking at patients holistically, or that patients were more than the sum of the variables used to predict risk. Senior nurses stressed that prediction models were only one part of patient evaluation, and clinicians should be encouraged to incorporate both model outputs and their own knowledge and experiences in decision making rather than trust models implicitly. Doctors also emphasised this holistic approach, adding that they placed more importance on hearing a nurse was concerned for the patient than seeing the model output. Critical thinking about future management was frequently raised in this context, with both nurses and doctors insisting that model predictions and the information required for contextualising risk scores should be communicated together when escalating the patient’s care to more senior clinicians.

### Model outputs

Model outputs were discussed in two contexts. First, doctors perceived that ordinal risk scores generated by Q-ADDS felt arbitrary compared to receiving probabilities of a future event, for example cardiorespiratory decompensation, that required a response such as resuscitation or high-level treatment. However, nurses did not wholly embrace probabilities as outputs, instead suggesting that recommendations for how they should respond to different Q-ADDS scores were more important. This difference may reflect the different roles of alert receivers (nurses) and alert responders (doctors).

*“[It’s helpful] if you use probabilities… If your patient has a sedation score of 2 and a respiratory rate of 10*,* [giving them] a probability of respiratory depression would be helpful. However*,* I don’t find many clinicians*,* and certainly beginning practitioners*,* think in terms of probabilities.”* – Clinical nurse consultant.

Second, there was frequent mention of alert fatigue in the context of model outputs. One doctor and two nurses felt there was insufficient leeway for nurses to exercise discretion in responding to risk scores, leading to many unnecessary alert-initiated actions. More nuance in the way Q-ADDS outputs were delivered to clinicians with different roles was deemed important to avoid model alerts being perceived as repetitive and unwarranted. However, three other doctors warned against altering MET call criteria in response to repetitive and seemingly unchanging risk scores and that at-risk patients should, as a standard of care, remain under frequent observation. Frustrations centred more often around rigidly tying repetitive Q-ADDS outputs to certain mandated actions, leading to multiple clinical reviews in a row for a patient whose trajectory was predictable, for example a patient with stable heart failure having a constantly low blood pressure. This led to duplication of nursing effort (e.g., repeatedly checking the blood pressure) and the perception that prediction models were overly sensitive.

*“It takes away a lot of nurses’ critical judgement. If someone’s baseline systolic [blood pressure] is 95 [mmHg]*,* they’re asymptomatic and I would never hear about it previously. We’re all aware that this is where they sit and that’s fine. Now they are required to notify me in the middle of the night*,* “Just so you know*,* they’ve dropped to 89 [below an alert threshold of 90mmHg].“”* – Junior doctor.

### Validation of clinical intuition

Clinicians identified the ability of prediction models to validate their clinical intuition as both a benefit and a hindrance, depending on how outputs were interpreted and acted upon. Junior clinicians appreciated early warning scores giving them more support to escalate care to senior clinicians, as a conversation starter or framing a request for discussion. Clinicians described how assessing the patient holistically first, then obtaining model outputs to add context and validate their diagnostic hypotheses, was very useful in deciding what care should be initiated and when.

*“You kind of rule [hypotheses] out… you go to the worst extreme: is it something you need to really be concerned about*,* especially if their [score] is quite high? You’re thinking of common complications like blood clots*,* so that presents as tachycardic… I’m thinking of a PE [pulmonary embolism]*,* then you do the nursing interventions.” –* Clinical nurse manager.

While deterioration alerts were often seen as triggers to think about potential causes for deterioration, participants noted that decision making could be compromised if clinicians were primed by model outputs to think of different diagnoses before they had fully assessed the patient at the bedside. Clinicians described the dangers of tunnel vision or, before considering all available clinical information, investigating favoured diagnoses to the exclusion of more likely causes.

*“[Diagnosis-specific warnings are] great*,* [but] that’s one of those things that can lead to a bit of confirmation bias… It’s a good trigger to articulate*,* “I need to look for sources of infection when I go to escalate"… but then*,* people can get a little bit sidetracked with that and ignore something more blatant in front of them. I’ve seen people go down this rabbit warren of being obsessed with the “fact” that it was sepsis*,* but it was something very*,* very unrelated.” –* Nurse educator.

### Objective measurement

Clinicians perceived that prediction models were useful as more objective measures of patients’ clinical status that could ameliorate clinical uncertainty or mitigate cognitive biases. In contrast to the risk of confirmation bias arising from front-loading model outputs suggesting specific diagnoses, prediction models could offer a second opinion that could help clinicians recognise opposing signals in noisy data that, in particular, assisted in considering serious diagnoses that shouldn’t be missed (e.g., sepsis), or more frequent and easily treated diagnoses (e.g., dehydration). Prediction models were also useful when they disclosed several small, early changes in patient status that provided an opportunity for early intervention.

*“Maybe [the patient has] a low grade fever*,* they’re a bit tachycardic. Maybe [sepsis] isn’t completely out of the blue for this person. If there was some sort of tool*,* that said there’s a reasonable chance that they could have sepsis here*,* I would use that to justify the option of going for blood cultures and maybe a full septic screen. If [I’m indecisive]*,* that sort of information could certainly push me in that direction.” –* Junior doctor.

Clinicians frequently mentioned that prediction models would have been more useful when first starting clinical practice, but become less useful with experience. However, clinicians noted that at any experience level, risk scoring was considered most useful as a triage/prioritisation tool, helping decide which patients to see first, or which clinical concerns to address first.

*“[Doctors] can easily triage a patient who’s scoring 4 to 5 versus 1 to 3. If they’re swamped*,* they can change the escalation process*,* or triage appropriately with better communication.”* – Clinical nurse manager.

Clinicians also stressed that predictions were not necessarily accurate because measurement error or random variation, especially one-off outlier values for certain variables, was a significant contributor to false alerts and inappropriate responses. For example, a single unusually high respiratory rate generated an unusually high risk score, prompting an unnecessary alert.

### Over-protocolisation of care

The sentiment most commonly expressed by all experienced nursing participants and some doctors was that nurses were increasingly being trained to solely react to model outputs with fixed response protocols, rather than think critically about what is happening to patients and why. It was perceived that prediction models may actually reduce the capacity for clinicians to process and internalise important information. For example, several nurses observed their staff failing to act on their own clinical suspicions that patients were deteriorating because the risk score had not exceeded a response threshold.

*“We’ve had patients on the ward that have had quite a high tachycardia*,* but it’s not triggering because it’s below the threshold to trigger… [I often need to make my staff] make the clinical decision that they can call the MET anyway*,* because they have clinical concern with the patient.” –* Clinical nurse consultant.

A source of great frustration for many nurses was the lack of critical thinking by their colleagues of possible causes when assessing deteriorating patients. They wanted their staff to investigate whether early warning score outputs or other changes in patient status were caused by simple, easily fixable issues such as fitting the oxygen mask properly and helping the patient sit up to breathe more easily, or whether they indicated more serious underlying pathophysiology. Nurses repeatedly referenced the need for clinicians to always be asking why something was happening, not simply reacting to what was happening.

*“[Models should also be] trying to get back to critical thinking. What I’m seeing doesn’t add up with the monitor*,* so I should investigate further than just simply calling the code.” –* Clinical nurse educator.

### Model transparency and interactivity

Clinicians frequently requested more transparent and interactive prediction models. These included a desire to receive more training in how prediction models worked and how risk estimates were generated mathematically, and being able to visualise important predictors of deterioration and the absolute magnitude of their effects (effect sizes) in intuitive ways. For example, despite receiving training in Q-ADDS, nurses expressed frustrations that nobody at the hospital seemed to understand how it worked in generating risk scores. Doctors were interested in being able to visualise the relative size and direction of effect of different model variables, potentially using colour-coding, combined with other contextual patient data like current vital sign trends and medications, and presented on one single screen.

The ability to modify threshold values for model variables and see how this impacted risk scores, and what this may then mean for altering MET calling criteria, was also discussed. For example, in an older patient with an acute ischaemic stroke, a persistently high, asymptomatic blood pressure value is an expected bodily response to this acute insult over the first 24–48 h. In the absence of any change to alert criteria, recurrent alerts would be triggered which may encourage overtreatment and precipitous lowering of the blood pressure with potential to cause harm. Altering the criteria to an acceptable or “normal” value for this clinical scenario (i.e. a higher than normal blood pressure) may generate a lower, more patient-centred risk estimate and less propensity to overtreat. This ability to tinker with the model may also enhance understanding of how it works.

*“I wish I could alter criteria and see what the score is after that*,* with another set of observations. A lot of the time… I wonder what they’re sitting at*,* now that I’ve [altered] the bit that I’m not concerned about… It would be quite helpful to refresh it and have their score refreshed as the new score.” –* Junior doctor.

### Derivation of the decision-making model

**Fig. 2:**
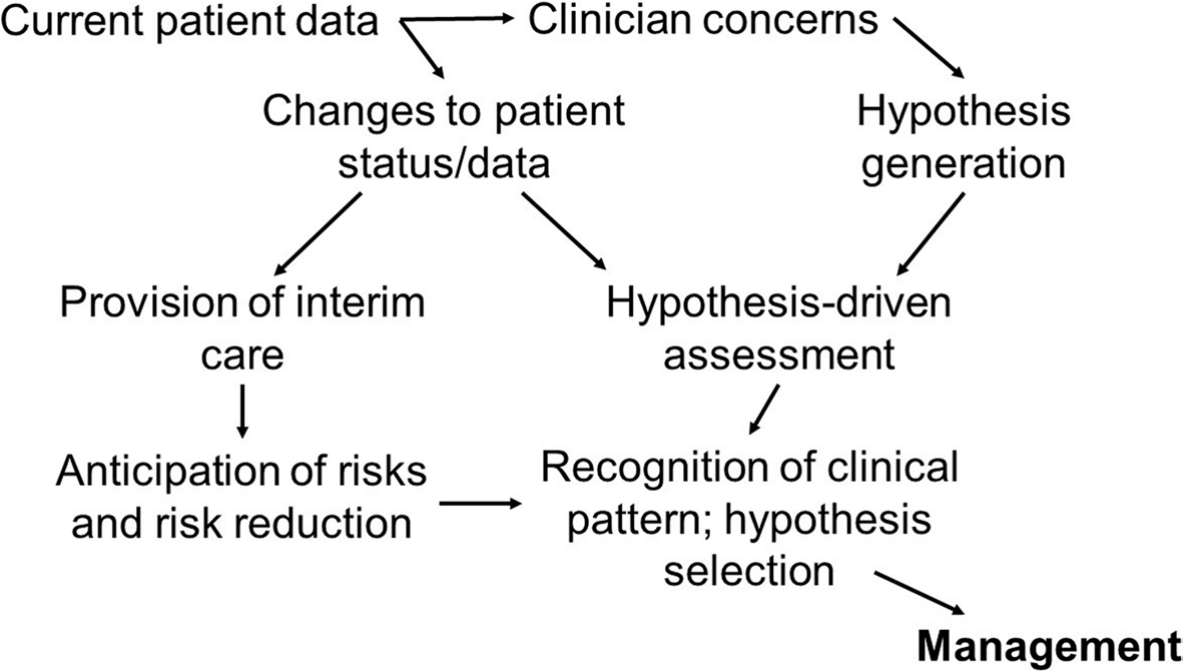
Decision-making model(Adapted from Neill’s clinical decision making framework [2005] and modified by Banning [2006]) with sequential decision nodes

Guided by the responses of our participants regarding their decision-making processes, our literature search identified a narrative review by Banning (2008) that reported previous work by O’Neill et al. (2005) [28, 29]. While these studies referred to models of nurse decision-making, we selected a model (Fig. 2) that also appropriately described the responses of doctors in our participant group and matched the context of using clinical decision support systems to support clinical judgement. As an example, when clinicians referenced needing to look for certain data points to give context to a patient assessment, this was mapped to nodes relating to “Current patient data,” “Changes to patient status/data,” and “Hypothesis-driven assessment.”

### Mapping of themes to decision-making model

**Fig. 3:**
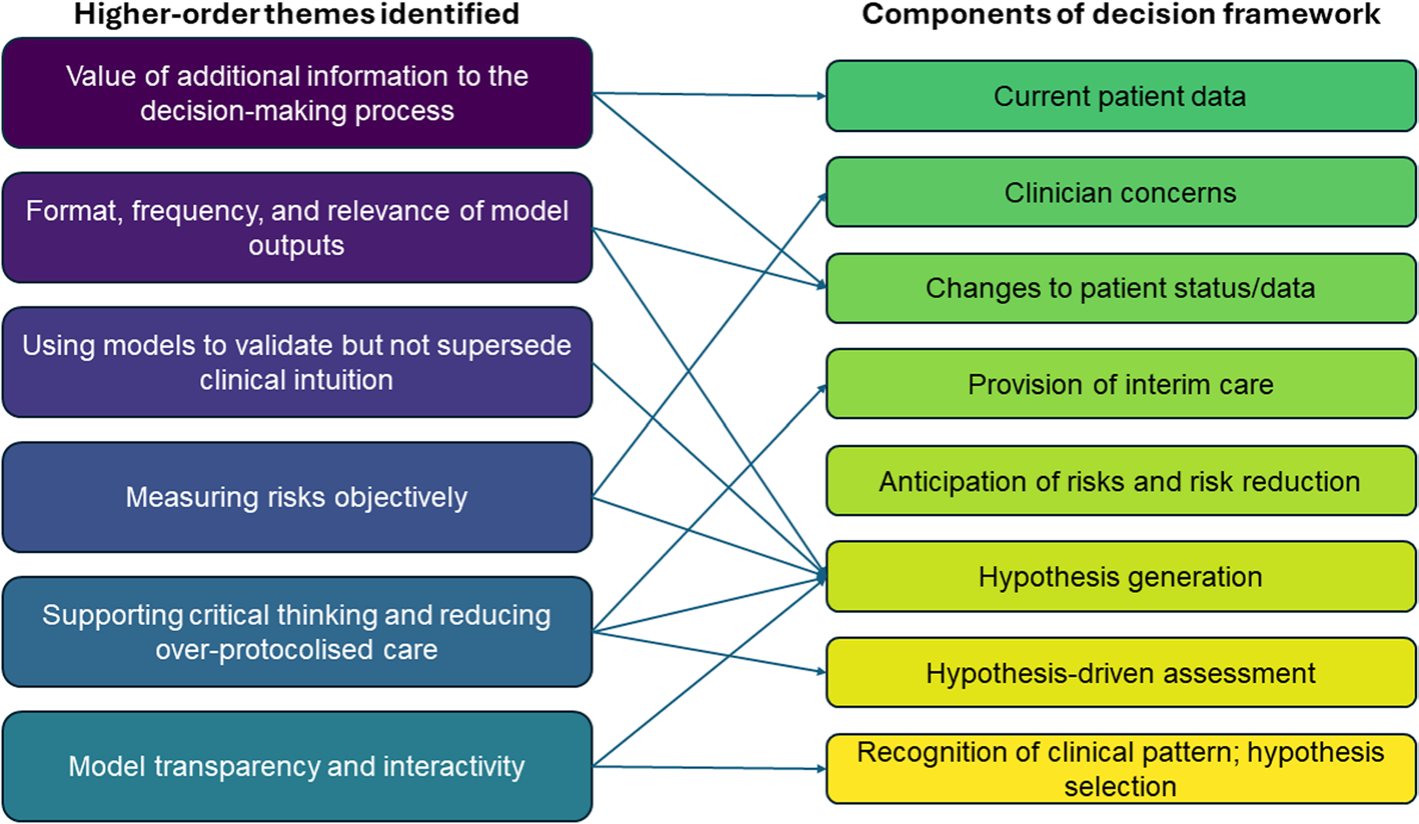
Mapping of the perceived relationships between higher-order themes and nodes in the decision-making model shown in Fig. 2

The themes from Table 1 were mapped to the nodes in the decision-making model based on close alignment with participant responses (see Fig. 3). This mapping is further explained below, where the nodes in the model are described in parentheses.

- *Value of additional information for decision-making*: participants stressed the importance of understanding not only the data going into the prediction model, but also how that data changed over time as trends, and the data that were not included in the model. (Current patient data, changes to patient status/data)
- *Format, frequency, and relevance of outputs*: participants suggested a change in patient data should not always lead to an alert. Doctors, but not necessarily nurses, proposed outputs displayed as probabilities rather than scores, tying model predictions to potential diagnoses or prognoses. (Changes to patient status/data, hypothesis generation)
- *Using models to validate but not supersede clinical intuition*: Depending on the exact timing of model outputs within the pathway of patient assessment, participants found predictions could either augment or hinder the hypothesis generation process. (Hypothesis generation)
- *Measuring risks objectively*: Risk scores can assist with triaging or prioritising patients by urgency or prognostic risk, thereby potentially leading to early intervention to identify and/or prevent adverse events. (Clinician concerns, hypothesis generation)
- *Supporting critical thinking and reducing over-protocolised care*: by acting as triggers for further assessment, participants suggested prediction models can support or discount diagnostic hypotheses, lead to root-cause identification, and facilitate interim cares, for example by ensuring good fit of nasal prongs. (Provision of interim care, hypothesis generation, hypothesis-driven assessment)
- *Model transparency and interactivity*: understanding how prediction models worked, being able to modify or add necessary context to model predictions, and understanding the relative contribution of different predictors could better assist the generation and selection of different hypotheses that may explain a given risk score. (Hypothesis generation, recognition of clinical pattern and hypothesis selection)

### Recommendations for improving the design of prediction models

Based on the mapping of themes to the decision-making model, we formulated four recommendations for enhancing the development and deployment of prediction models for clinical deterioration.

1. Improve accessibility and transparency of data included in the model. Provide an interface that allows end-users to see what predictor variables are included in the model, their relative contributions to model outputs, and facilitate easy access to data not included in the model but still relevant for model-informed decisions, e.g., trends of predictor variables over time.
2. Present model outputs that are relevant to the end-user receiving those outputs, their responsibilities, and the tasks they may be obliged to perform, while preserving the ability of clinicians to apply their own discretionary judgement.
3. In situations associated with diagnostic uncertainty, avoid tunnel vision from priming clinicians with possible diagnostic explanations based on model outputs, prior to more detailed clinical assessment of the patient.
4. Support critical thinking whereby clinicians can apply a more holistic view of the patient’s condition, take all relevant contextual factors into account, and be more thoughtful in generating and selecting causal hypotheses.

## Discussion

This qualitative study involving front-line acute care clinicians who respond to early warning score alerts has generated several insights into how clinicians perceive the use of prediction models for clinical deterioration. Clinicians preferred models that facilitated critical thinking, allowed an understanding of the impact of variables included and excluded from the model, provided model outputs specific to the tasks and responsibilities of different disciplines of clinicians, and supported decision-making processes in terms of hypotheses and choice of management, rather than simply responding to alerts in a pre-specified, mandated manner. In particular, preventing prediction models from supplanting critical thinking was repeatedly emphasised.

Reduced staffing ratios, less time spent with patients, greater reliance on more junior workforce, and increasing dependence on automated activation of protocolised management are all pressures that could lead to a decline in clinical reasoning skills. This problem could be exacerbated by adding yet more predictive algorithms and accompanying protocols for other clinical scenarios, which may intensify alert fatigue and disrupt essential clinical care. However, extrapolating our results to areas other than clinical deterioration should be done with caution. An opposing view may be that using prediction models to reduce the burden of routine surveillance may allow redirection of critical thinking skills towards more useful tasks, a question that has not been explored in depth in the clinical informatics literature.

Clinicians expressed interest in models capable of providing causal insights into clinical deterioration. This is neither a function nor capability of most risk prediction models, requiring different assumptions and theoretical frameworks [30]. Despite this limitation, risk nomograms, visualisations of changes in risk with changes in predictor variables, and other interactive tools for estimating risk may be useful adjuncts for clinical decision-making due to the ease with which input values can be manipulated.

### Contributions to the literature

Our research supports and extends the literature on the acceptability of risk prediction models within clinical decision support systems. Common themes in the literature supporting good practices in clinical informatics and which are also reflected in our study include: alert fatigue; the delivery of more relevant contextual information; [31] the value of patient histories; [32, 33] ranking relevant information by clinical importance, including colour-coding; [34, 35] not using computerised tools to replace clinical judgement; [32, 36, 37] and understanding the analytic methods underpinning the tool [38]. One other study has investigated the perspectives of clinicians of relatively simple, rules-based prediction models similar to Q-ADDS. Kappen et al [12] conducted an impact study of a prediction model for postoperative nausea and vomiting and also found that clinicians frequently made decisions in an intuitive manner that incorporated information both included and absent from prediction models. However, the authors recommended a more directive than assistive approach to model-based recommendations, possibly due to a greater focus on timely prescribing of effective prophylaxis or treatment.

The unique contribution of our study is a better understanding of how clinicians may use prediction models to generate and validate diagnostic hypotheses. The central role of critical thinking and back-and-forth interactions between clinician and model in our results provide a basis for future research using more direct investigative approaches like cognitive task analysis [39]. Our study has yielded a set of cognitive insights into decision making that can be applied in tandem with statistical best practice in designing, validating and implementing prediction models. [19, 40, 41]. 

### Relevance to machine learning and artificial intelligence prediction models for deterioration

Our results may generalise to prediction models based on machine learning (ML) and artificial intelligence (AI), according to results of several recent studies. Tonekaboni et al [42] investigated clinician preferences for ML models in the intensive care unit and emergency department using hypothetical scenarios. Several themes appear both in our results and theirs: a need to understand the impact of both included and excluded predictors on model performance; the role of uncertain or noisy data in prediction accuracy; and the influence of trends or patient trajectories in decision making. Their recommendations for more transparent models and the delivery of model outputs designed for the task at hand align closely with ours. The authors’ focus on clinicians’ trust in the model was not echoed by our participants.

Eini-Porat et al [43] conducted a comprehensive case study of ML models in both adult and paediatric critical care. Their results present several findings supported by our participants despite differences in clinical environments: the value of trends and smaller changes in several vital signs that could cumulatively signal future deterioration; the utility of triage and prioritisation in time-poor settings; and the use of models as triggers for investigating the cause of deterioration.

As ML/AI models proliferate in the clinical deterioration prediction space, [44] it is important to deeply understand the factors that may influence clinician acceptance of more complex approaches. As a general principle, these methods often strive to input as many variables or transformations of those variables as possible into the model development process to improve predictive accuracy, incorporating dynamic updating to refine model performance. While this functionality may be powerful, highly complex models are not easily explainable, require careful consideration of generalisability, and can prevent clinicians from knowing when a model is producing inaccurate predictions, with potential for patient harm when critical healthcare decisions are being made [45–47]. Given that our clinicians emphasised the need to understand the model, know which variables are included and excluded, and correctly interpret the format of the output, ML/AI models in the future will need to be transparent in their development and their outputs easily interpretable.

### Limitations

The primary limitations of our study were that our sample was drawn from two hospitals with high levels of digital maturity in a metropolitan region of a developed country, with a context specific to clinical deterioration. Our sample of 15 participants may be considered small but is similar to that of other studies with a narrow focus on clinical perspectives [42, 43]. All these factors can limit generalisability to other settings or to other prediction models. As described in the methods, we used open-ended interview templates and generated our inductive themes reflexively, which is vulnerable to different types of biases compared to more structured preference elicitation methods with rigidly defined analysis plans. Member checking may have mitigated this bias, but was not possible due to the time required from busy clinical staff.

Our study does not directly deal with methodological issues in prediction model development, [41, 48] nor does it provide explicit guidance on how model predictions should be used in clinical practice. Our findings should also not be considered an exhaustive list of concerns clinicians have with prediction models for clinical deterioration, nor may they necessarily apply to highly specialised clinical areas, such as critical care. Our choice of decision making framework was selected because it demonstrated a clear, intuitive causal pathway for model developers to support the clinical decision-making process. However, other, equally valid frameworks may have led to different conclusions, and we encourage more research in this area.

## Conclusion

This study elicited clinician perspectives of models designed to predict and manage impending clinical deterioration. Applying these perspectives to a decision-making model, we formulated four recommendations for the design of future prediction models for deteriorating patients: improved transparency and interactivity, tailoring models to the tasks and responsibilities of different end-users, avoiding priming clinicians with diagnostic predictions prior to in-depth clinical review, and finally, facilitating the diagnostic hypothesis generation and assessment process.

### Supplementary Information

Supplementary Material 1.

## Data Availability

Due to privacy concerns and the potential identifiability of participants, interview transcripts are not available. However, interview guides are available in the supplement.
