# Supplementary material for: Clinician perspectives and recommendations regarding design of clinical prediction models for deteriorating patients in acute care
Source: BMC Med Inform Decis Mak. 2024 Sep 2;24:241. doi: 10.1186/s12911-024-02647-4 (PMC11367817; doi:10.1186/s12911-024-02647-4)
Supplement: Supplementary file 1 — Supplementary Material 1. [file 12911_2024_2647_MOESM1_ESM.docx]

**Supplementary file**

**Participant information**

| **Cumulative years of practice** | **Nurses** | **Doctors** |
| --- | --- | --- |
| 0 to 2 | 2 | 3 |
| 2 to 5 | 1 | 1 |
| 5 to 10 | 1 | 2 |
| 10 or more | 4 | 1 |
| **Speciality** |  |  |
| General surgery | 0 | 1 |
| Intern | 0 | 2 |
| Nurse education | 1 | 0 |
| MAPU | 1 | 0 |
| Orthopaedics | 2 | 0 |
| Oncology | 1 | 0 |
| Registrar | 0 | 2 |
| Resident | 0 | 1 |
| None/other | 3 | 1 |
| **Gender** |  |  |
| Male | 1 | 5 |
| Female | 7 | 2 |

*MAPU: Medical Assessment and Planning Unit*

**Queensland Adult Deterioration of Deterioration System**

The Q-ADDS, developed by Queensland Department of Health, comprises a cumulative scoring system in that MET alert thresholds for both an aggregate score across multiple parameters, or severe derangement on a single parameter, can be used to activate a MET call.

An aggregate score ≥8 or any single parameter that reaches the E (emergency) threshold will activate a MET call:

| Parameter | Score |
| --- | --- |
| Respiratory rate | 1: 9-12 or 21-24  2: 25-29  4: 31-35  E: ≥ 36 |
| Oxygen saturation (%) | 1: 90-94%  2: 85-89%  4: ≤ 84% |
| Oxygen delivery (L/min or % delivered) | 1: 2-5L/min OR 28-40%  2: >5-11L/min OR >40- 50%  4: 11-14L/min OR ≥50% - 59%  E: 15L/min OR ≥ 60% |
| Blood Pressure – Systolic (mmHg) | 1: 100-109 OR 160-169 mmHg  2: 90-99 OR 170-199mmHg  4: 80–89 OR ≥200 mmHg  E: <80mmHg |
| Heart Rate (beats/min) | 1: 100-109 bpm  2: 40-49 OR 110-129 bpm  3: 130-139 bpm  4: 140-159 bpm  E: < 40 bpm or ≥160bpm |
| Temperature (°C) | 1: 35.1-36.0 OR 38 to 38.4  2: 34.1-35.0 OR 38.5 to ≥ 39.5  4: ≤ 34 |
| Consciousness (AVPU) | 1: Responds to Voice  E: Responds only to Pain or Unresponsive |
| New confusion/agitation | 4: if present |

**Interview guides**

Interview guides were rough outlines only, meant to set the scene for discussion between interviewers and participants. Tangents were frequently allowed to be explored to ensure that sufficient information on CPMs could be investigated. The nurse and doctor interview guides provided below were not used for all interviews, and not all questions were explored.

**Nursing interview guide**

**Understanding the information nurses want from clinical deterioration alerts**

**How to use this guide:**

This guide is based on the domains of the non-adoption, abandonment, scale-up, spread, sustainability (NASSS) framework^1^ to ensure all aspects of digital health implementation are considered. Questions are adapted from the recently published NASS-CAT INTERVIEW guide^2^. It is divided into sections based on the participant’s role as a technology developer/designer, organizational lead, technology user, organizational horizon scanner and clinical specialist. The interviewer should follow the questions and prompts from the section(s) of the guide that most appropriately match the interviewees’ role(s). Within each section:

- - 1. All key questions should be asked
    2. Should other relevant issues be raised they should be explored using the prompts as required
    3. Interviewers will ensure discussion progresses in a timely, yet informative manner

References

1. Greenhalgh T, Wherton J, Papoutsi C, Lynch J, Hughes G, Hinder S, et al. Beyond adoption: a new framework for theorizing and evaluating nonadoption, abandonment, and challenges to the scale-up, spread, and sustainability of health and care technologies. Journal of medical Internet research. 2017;19(11):e367
2.
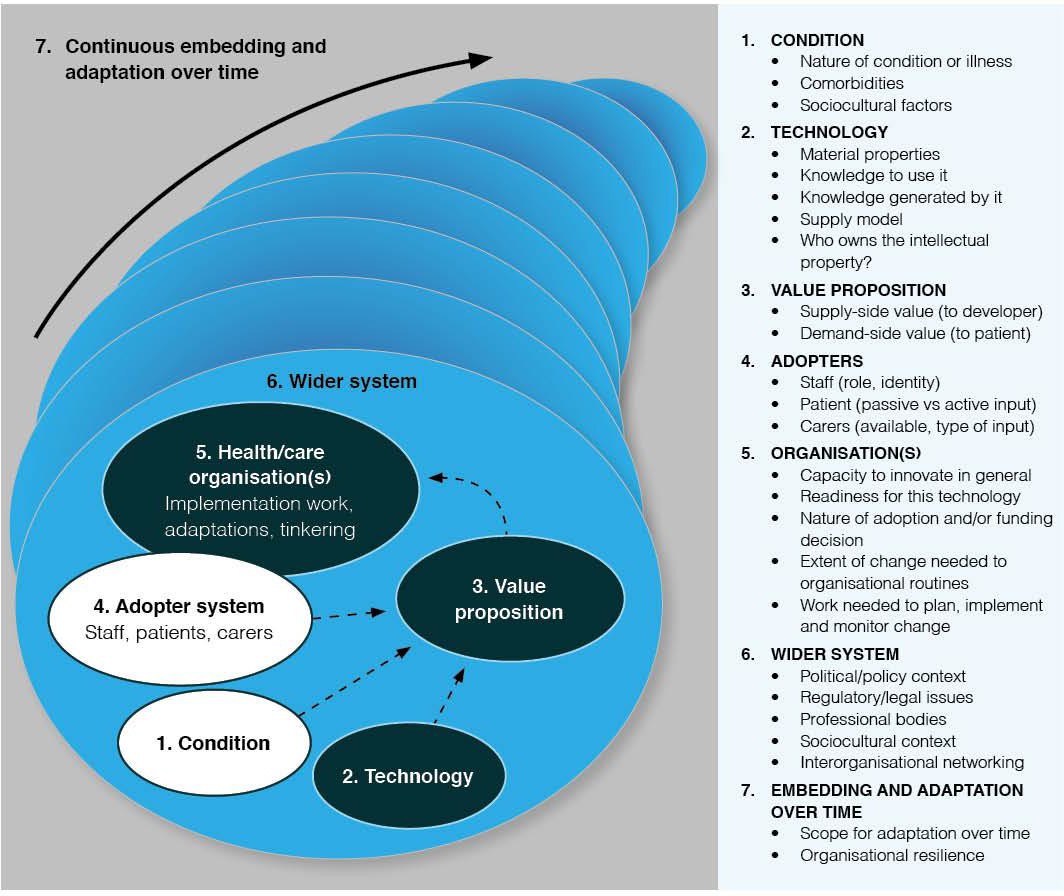
Greenhalgh T, Maylor H, Shaw S, Wherton J, Papoutsi C, Betton V, Nelissen N, Gremyr A, Rushforth A, Koshkouei M, Taylor J. The NASSS-CAT Tools for Understanding, Guiding, Monitoring, and Researching Technology Implementation Projects in Health and Social Care: Protocol for an Evaluation Study in Real-World Settings. JMIR Research Protocols. 2020;9(5):e16861.

*1 Diagram: The NASSS framework (© Greenhalgh at al J Med Internet Research 2017; 19 (11): e367)*

**Introduction**

Hi, my name is [ ]. I’m speaking with you today because we want to understand how we can improve automated alerts for clinically deteriorating patients. Your name(s) was given as a key stakeholder, and we believe you have a valuable perspective to share with us due to your role in managing patients.

We will record the conversation with you, if that is OK, and will only share the recording with other members of our research team. We will ensure that nobody outside the research team will be able to identify you through your responses. We really appreciate your time and willingness to speak with us. Do you have any questions before we begin?

**Context**

**1. Can you tell me about your role as a clinician(s) managing deteriorating patients?**

- How long have you been a clinician for?

- How often do you participate in MET (Medical Emergency Team) calls?

**2. Can you tell me what you need to do when you get a deterioration alert, or perceive a patient is deteriorating?**

- Can you give me a recent example of this process?

- For example, a checklist you follow?

**3. What would you consider some of the barriers you face when you are helping to manage or escalate a deteriorating patient?**

- Can you give me some concrete examples?

- Why do you think this is the case?

- Has this changed over time?

**4. Can you tell me whether deterioration alerts help you better manage your patients?**

- Do you find the alerts unhelpful or unnecessary extra work?

- Did the alerts give you confidence to escalate the patient’s care, or help you decide faster?

**Technology and Adopters**

**5. Let’s talk about the things that you believe could best support you to better manage clinical deterioration. If we could give you any information you wanted, what would you find valuable?**

- Are there things we could pull from the patient’s chart to improve or speed up your workflow?

- What about automatically including a checklist for common issues like sepsis?

**6. I want to delve a bit deeper into understanding how useful you might find these insights. Let’s say we set up a statistical model to predict whether a patient was going to become septic, or experience acute kidney problems. Would this information be useful?**

- Would you want to see which variables led to that prediction?

- Would you trust these predictions?

- Would these predictions help with your workload?

**Value Proposition**

**7. Last question. What would be the benefit of the technologies we discussed?**

- How much time do you think these decision support tools might save you?

- What do you think would be the benefit for patients?

**[End of Interview]**

**Doctor interview guide**

**Understanding the information doctors want from clinical deterioration alerts**

**How to use this guide:**

This guide is based on the domains of the non-adoption, abandonment, scale-up, spread, sustainability (NASSS) framework^1^ to ensure all aspects of digital health implementation are considered. Questions are adapted from the recently published NASS-CAT INTERVIEW guide^2^. It is divided into sections based on the participant’s role as a technology developer/designer, organizational lead, technology user, organizational horizon scanner and clinical specialist. The interviewer should follow the questions and prompts from the section(s) of the guide that most appropriately match the interviewees’ role(s). Within each section:

- - 1. All key questions should be asked
    2. Should other relevant issues be raised they should be explored using the prompts as required
    3. Interviewers will ensure discussion progresses in a timely, yet informative manner

References

1. Greenhalgh T, Wherton J, Papoutsi C, Lynch J, Hughes G, Hinder S, et al. Beyond adoption: a new framework for theorizing and evaluating nonadoption, abandonment, and challenges to the scale-up, spread, and sustainability of health and care technologies. Journal of medical Internet research. 2017;19(11):e367
2.
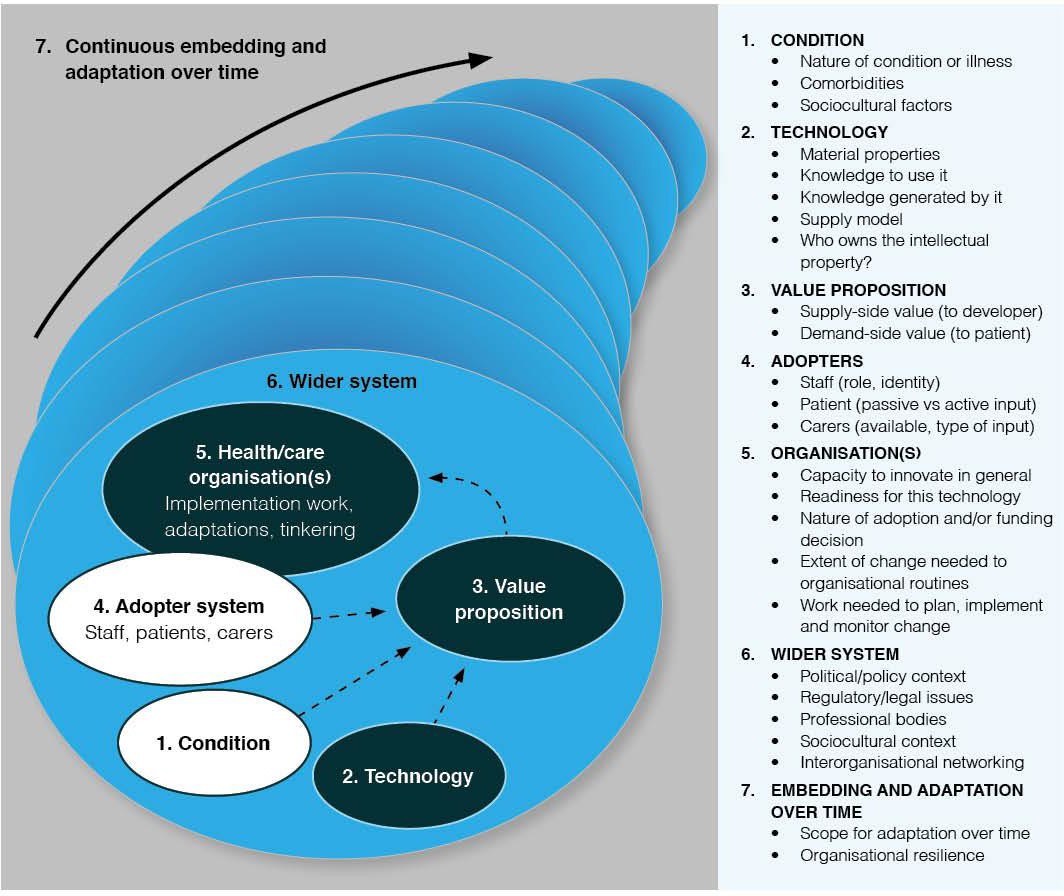
Greenhalgh T, Maylor H, Shaw S, Wherton J, Papoutsi C, Betton V, Nelissen N, Gremyr A, Rushforth A, Koshkouei M, Taylor J. The NASSS-CAT Tools for Understanding, Guiding, Monitoring, and Researching Technology Implementation Projects in Health and Social Care: Protocol for an Evaluation Study in Real-World Settings. JMIR Research Protocols. 2020;9(5):e16861.

*1 Diagram: The NASSS framework (© Greenhalgh at al J Med Internet Research 2017; 19 (11): e367)*

**Introduction**

Hi, my name is [ ]. I’m speaking with you today because we want to understand how we can improve automated alerts for clinically deteriorating patients. Your name(s) was given as a key stakeholder, and we believe you have a valuable perspective to share with us due to your role in managing patients.

We will record the conversation with you, if that is OK, and will only share the recording with other members of our research team. We will ensure that nobody outside the research team will be able to identify you through your responses. We really appreciate your time and willingness to speak with us. Do you have any questions before we begin?

**Context**

**1. Can you tell me about your role as a clinician(s) managing deteriorating patients?**

- How long have you been practicing for?

- How often do deterioration-related events happen? Daily?

**2. Can you tell me what you need to do when you are notified or perceive that a patient is deteriorating?**

- Can you give me a recent example of this process? For example, is there a formal or informal checklist you follow?

**3. What would you consider some of the main knowledge barriers you encounter when you are managing a deteriorating patient?**

- Can you give me some concrete examples?

- Why do you think this is the case?

- Has this changed over time?

**4. Can you tell me whether deterioration alerts, for example the Q-ADDS, help you better manage your patients?**

- How do your care plans change when a patient is deteriorating?

- What do you think would expedite that process of finding a better management plan for a given patient?

**Technology and Adopters**

**5. Ok, I’d like us to now take a step back and talk about the things that you believe could best support you to better manage clinical deterioration in an ideal world. If we could give you any information you wanted, what would you find valuable?**

- Are there things we could pull from the patient’s chart? Or predictive measures we could provide?

- What about automatically including a checklist or set of criteria for common issues like sepsis?

**6. I want to delve a bit deeper into understanding how useful you might find these insights. Let’s say we set up a statistical model that worked in the background to predict whether a patient was going to become septic, or experience acute kidney problems. To what extent do you think this information would help?**

- Would you want to see which variables led to that prediction?

- Would you trust these predictions?

- Would these predictions help with your workload?

**Value Proposition**

**7. Last question. What would be the benefit of the information technologies we discussed?**

- How much time do you think these decision support tools might save you?

- What do you think would be the benefit for patients?

**[End of Interview]**
